# Individualized and institutionalized residential place-based discrimination and self-rated health: a cross-sectional study of the working-age general population in Osaka city, Japan

**DOI:** 10.1186/1471-2458-14-449

**Published:** 2014-05-13

**Authors:** Takahiro Tabuchi, Tomoki Nakaya, Wakaba Fukushima, Ichiro Matsunaga, Satoko Ohfuji, Kyoko Kondo, Miki Inui, Yuka Sayanagi, Yoshio Hirota, Eiji Kawano, Hiroyuki Fukuhara

**Affiliations:** 1 Center for Cancer Control and Statistics, Osaka Medical Center for Cancer and Cardiovascular Diseases, 3-3 Nakamichi 1-Chome, Higashinari-ku, Osaka, 537-8511, Japan; 2 Urban Research Plaza, Osaka City University, 3-3-138 Sugimoto, Sumiyoshi-ku, Osaka 558-8585, Japan; 3 Department of Geography, College of Letters, Ritsumeikan University, 56-1 Tojiin-kita-machi, Kita-ku, Kyoto 603-8577, Japan; 4 Department of Public Health, Osaka City University Faculty of Medicine, 1-4-3, Asahi-machi, Abeno-ku, Osaka 545-8585, Japan; 5 Department of Sociology, Osaka City University, 3-3-138 Sugimoto, Sumiyoshi-ku, Osaka 558-8585, Japan; 6 Department of Economics, Osaka City University, 3-3-138 Sugimoto, Sumiyoshi-ku, Osaka 558-8585, Japan

**Keywords:** Place-based discrimination, Self-rated health, Osaka city in Japan, Multilevel analysis, Individualized and institutionalized pathways

## Abstract

**Background:**

Several studies have reported that individualized residential place-based discrimination (PBD) affects residents’ health. However, studies exploring the association between institutionalized PBD and health are scarce, especially in Asian countries including Japan.

**Methods:**

A cross-sectional study was conducted with random two-stage sampling of 6191 adults aged 25–64 years in 100 census tracts across Osaka city in 2011. Of 3244 respondents (response rate 52.4%), 2963 were analyzed using multilevel logistic regression to examine the association of both individualized and institutionalized PBD with self-rated health (SRH) after adjustment for individual-level factors such as socioeconomic status (SES). An area-level PBD indicator was created by aggregating individual-level PBD responses in each tract, representing a proxy for institutionalized PBD, i.e., the concept that living in a stigmatized neighborhood affects neighborhood health. 100 tracts were divided into quartiles in order. The health impact of area-level PBD was compared with that of area-level SES indicators (quartile) such as deprivation.

**Results:**

After adjustment for individual-level PBD, the highest and third area-level PBD quartiles showed odds ratio (OR) 1.57 (95% credible interval: 1.13-2.18) and 1.38 (0.99-1.92), respectively, for poor SRH compared with the lowest area-level PBD quartile. In a further SES-adjusted model, ORs of area-level PBD (highest and third quartile) were attenuated to 1.32 and 1.31, respectively, but remained marginally significant, although those of the highest area-level not-home-owner (census-based indicator) and deprivation index quartiles were attenuated to 1.26 and 1.21, respectively, and not significant. Individual-level PBD showed significant OR 1.89 (1.33-2.81) for poor SRH in an age, sex, PBD and SES-adjusted model.

**Conclusion:**

Institutionalized PBD may be a more important environmental determinant of SRH than other area-level SES indicators such as deprivation. Although it may have a smaller health impact than individualized PBD, attention should be paid to invisible and unconscious aspects of institutionalized PBD to improve residents’ health.

## Background

Understanding the impact of place on health is a key element of epidemiologic investigation [1,2]. Various aspects of residential place, such as green space, walkability, social capital, poverty, unemployment and deprivation, have been studied as the environmental determinants of health [3-9]. The concept of an association between place and discrimination such as racial/ethnic residential segregation is also one of the most investigated research fields in the USA [10,11]. Residential segregation based on race is associated with poor health outcomes among African Americans in the USA. Economic segregation and poverty concentrations are closely linked with such residential segregation [10]. However, studies based on this concept outside the USA are scarce, especially in Asian countries. On the other hand, the concept of discrimination due to geographical place of residence (place-based discrimination; PBD) has been explored as “territorial stigmatization”. Within the fields of sociology and geography, this exploration has focused on the bottom level of the hierarchy of place, in areas such as St Paul’s in Bristol, UK and the *banlieues* in France [12,13].

In Japan, the Buraku people are a minority group that continues to face discrimination because their predecessors, the *eta* and *hinin*, were considered outcasts during the Japanese *Edo* period (1600–1868) when they were employed socially unacceptable jobs (such as slaughtering animals or treating leather). In present day Japan this discrimination occurs in many aspects of life, including employment, housing and education. Further, discrimination against Buraku district residents, which is based on the place itself regardless of residents’ ancestry, continues [14]. At the same time, a new type of discrimination against residents of the Nishinari ward in Osaka city, which includes the largest Buraku district, so-called *Nishinari* discrimination has emerged [14,15]. This refers to the stigmatization of the ward for reasons of slums, poverty, crime, dilapidated dwellings and insanitary conditions. These two forms of discrimination, Buraku and Nishinari, could be categorized as PBD, which may affect the health of individuals who live in the specified area [16].

Many studies of racial/ethnic residential segregation have compared residents in a segregated area with those in other areas [10,11]. However, some discrimination studies focused only on the bottom level of the hierarchy of place, such as our previous study [16]. We reported that interpersonal perceived PBD, such as *Buraku* or *Nishinari* discrimination [15,17], was associated with poor mental health in a stigmatized area in Osaka, Japan [16]. However, this previous study treated only the individual-level perceived discrimination (individualized pathway), which is unlikely to capture the full complexity of PBD [18]. It is therefore important, when considering the mechanisms between PBD and health, that both individualized and institutionalized pathways are taken into account [19,20]. PBD may not be merely the actions and prejudices of individuals against individuals; institutionalized PBD may be perpetuated by organizations and represent processes built into social entities, in a similar way to institutionalized racism [19]. Thus, even if residents do not recognize the existence of interpersonal PBD, they may suffer inherent institutionalized PBD and, in turn, their health may be compromised; i.e., the concept that living in a stigmatized neighborhood affects neighborhood health. Our objective was to examine the association of both individualized and institutionalized PBD with health, using the self-rated health (SRH) index, in Osaka city. This city has a wide ranging population from the very poor to the affluent of Japan and includes some stigmatized areas, identified from a priori knowledge of *Buraku* and *Nishinari* discrimination [9,14]. We focused on area-level contextual impact on health, comparing the health impact of institutionalized PBD with that of other area-level indicators relating to socioeconomic status (SES), after adjusting for individual-level factors including perceived PBD and SES.

## Methods

### Study participants

A cross-sectional study was conducted from September to November 2011 in Osaka, a city with a population of 2.7 million. We randomly selected 100 of the 1,759 census enumeration tracts (each tract has average of 1,500 inhabitants) of the city, and randomly sampled 63 adults in each tract from the governmental Basic Resident Register database, which included all Japanese residents who had address in the area [21]. After excluding inhabitants who had recently migrated (had an address outside the selected tracts), 6,191 adults aged 25–64 years as of August 1, 2011 were systematically selected. Self-administered questionnaires were distributed and collected by mail. We visited non-responders at least three times with at least one visit on a weekend or in the evening. For data quality control, missing or inconsistent answers were re-tested by telephone. 3,244 subjects were available and provided written consent, giving a response rate of 52.4%. The study was approved by the Ethics Committee of Osaka City University.

### Outcome variable

The outcome variable was poor SRH. The value of measuring SRH as a predictor of mortality risk has been extensively reported [22]. SRH was measured through the question “In general, what is your current health status: excellent, very good, good, fair or poor?” For analysis purposes, this variable was dichotomized according to the previous studies [23,24] as good (“excellent/very good/good”) and poor (“fair/poor”). SRH primarily captures physical health and, to some extent, mental health [25].

### Individual-level PBD

Participants were asked the following question to measure their individual-level perceived PBD: “How often have you suffered discrimination based on geographical place of residence in your personal or social life? (frequently, sometimes or never)”. This variable was dichotomized into yes (frequently and sometimes) and no (never).

### Area (tract)-level indicators

Six characteristics of place (tract) were measured by two methods. First, area-level aggregates of survey responses were used to characterize neighborhoods, as participants in each neighborhood are viewed as informants of the conditions in their area [26]; and second, Japanese census 2005 based measures. An area-level PBD (ALPBD) indicator was created by aggregating individual-level perceived PBD responses among each tract, representing a proxy for institutionalized PBD, i.e., the concept that living in a stigmatized neighborhood affects neighborhood health. The percentage of unemployed residents and the percentage of those who did not own their own house (according to both aggregates and census-based methods) were also used as area-level SES indicators, because these factors were available in the 2005 census and considered as representative social determinants of health [6,27-29]. Another area-level indicator was an area-level deprivation index (ALDI), which was constructed using the census 2005 data for unemployment, housing tenure, aging and poverty, with an adjusted range of 0 to 100. The details of how to construct this index and how it showed health disparity in Japan are given elsewhere (see Additional file 1) [8]. The 100 tracts were divided into quartiles; the higher quartiles represented the more disadvantaged neighborhoods.

### Individual-level covariates

The variables included in the adjustment model were age, sex, SES and social relationships. In terms of SES, working status, housing tenure and educational attainment were used. Working status was categorized as “working”, “not working including retired and housewife” or “unemployed”. Housing tenure was dichotomized as “home owner by self or household members” or “not”. Education attainment was dichotomized as “high school (12 years education or less)” or “college or more (more than 12 years education)”. Social relationships were categorized by “number of friends” and “marital status”. “Number of friends” was categorized as “0”, “1-4”, or “5 or more”. Marital status was dichotomized as “married” or “not”.

### Statistical analyses

Participants who had moved within the previous year (n = 263), or had missing mobility data (n = 12) or PBD (n = 7) were excluded, because recent migration was likely to cause misclassification for ALPBD. The remaining 2,963 subjects were analyzed. Each tract included between 14 and 44 subjects (Additional file 1: Table S1). Chi-squared tests were used to compare the difference in subjects’ characteristics according to perceived PBD and poor SRH. Basic statistics such as mean, median and Pearson correlation coefficients were calculated for assessing the association between area-level indicators before making quartiles.

Individuals (first-level) were nested in the districts (second-level). The analysis framework anticipated that individual health outcomes would be partly dependent on the districts where individuals live. We therefore used multilevel models to estimate the variation in outcome between districts (random effects) and the impact of area-level indicators on the outcome with adjustment for individual compositional characteristics (fixed effects). Multilevel logistic regression models with random intercepts and fixed slopes using Markov Chain Monte Carlo methods, with chain length 50,000 burn-in 5,000 [30], were applied using the MLwiN 2.25 software package (Centre for Multilevel Modeling, University of Bristol, UK). Each area-level characteristic was tested in a separate model because we started from a simple comparison between the area-level indicators and consider multiple area-level adjustment an issue for future research.

Firstly, unadjusted and age and sex-adjusted odds ratios (ORs) and 95% credible intervals (CIs) for poor SRH were calculated (model 1 and 2, respectively). To distinguish the individual-level compositional impact and area-level contextual impact of PBD (or SES) on outcome, a corresponding individual-level PBD (or SES) variable was added into model 3. To compare six area-level indicators after adjustment of the same covariates including individual-level PBD and SES, adjustments for all individual-level PBD and SES (working status, housing tenure and educational attainment) in addition to age and sex were conducted as a key model (model 4). As social relationships might possibly mediate the association between area-level PBD and health, further adjustments for social relationships were applied to evaluate those potential pathways (model 5, see Additional file 1).

Probability values for statistical tests were two-tailed and *P* <0.05 and *P* <0.1 were considered statistically significant and marginally significant, respectively. Descriptive analyses were performed using SAS version 9.2 (SAS Institute, Cary, NC, USA).

## Results

Basic characteristics of the study subjects and the proportions of perceived PBD and poor SRH are shown in Table 1. Women were more likely to answer “yes” to experiencing PBD than men. A higher proportion of perceived PBD was observed among subjects who were less educated, had no spouse or had friends. The proportion of poor SRH significantly differed for all factors. A higher proportion of poor SRH was observed among subjects who had low SES or low social relationships.

**Table 1:** Basic characteristics of subjects (n = 2963)

		**Subjects**	**Individual-level place-based discrimination, yes**		**Poor self-rated health**	
**Characteristics**		**N (%)**	**N (%)**	***P *** **for difference**^**a**^	**N (%)**	***P *** **for difference**^**a**^
Sex	Male	1332 (45.0)	61 (4.6)	<0.01	209 (15.7)	<0.01
	Female	1631 (55.0)	130 (8.0)		195 (12.0)	
Age group	25-34 years	660 (22.3)	36 (5.5)	0.06	57 (8.6)	<0.01
	35-44 years	820 (27.7)	50 (6.1)		94 (11.5)	
	45-54 years	680 (22.9)	59 (8.7)		107 (15.7)	
	55-65 years	803 (27.1)	46 (5.7)		146 (18.2)	
Perceived place-based discrimination	No	2772 (93.6)	NA		359 (13.0)	<0.01
	Yes	191 (6.5)	NA		45 (23.6)	
Working status	Working	2294 (77.4)	141 (6.2)	0.47	249 (10.9)	<0.01
	Not working	502 (17.0)	35 (7.0)		116 (23.1)	
	Unemployed	156 (5.3)	13 (8.3)		37 (23.7)	
	Missing	11 (0.4)	NA		NA	
Housing tenure	Home owner	1705 (57.5)	108 (6.3)	0.74	181 (10.6)	<0.01
	Not home owner	1250 (42.2)	83 (6.6)		221 (17.7)	
	Missing	8 (0.3)	NA		NA	
Education attainment	High school or less	1311 (44.3)	102 (7.8)	<0.01	232 (17.7)	<0.01
	College or more	1646 (55.6)	89 (5.4)		171 (10.4)	
	Missing	6 (0.2)	NA		NA	
Number of friends	0	284 (9.6)	8 (2.8)	0.02	72 (25.4)	<0.01
	1-4	1171 (39.5)	72 (6.2)		175 (14.9)	
	5 or more	1480 (50.0)	107 (7.2)		153 (10.3)	
	Missing	28 (0.9)	NA		NA	
Marital status	Married	1750 (59.0)	97 (5.5)	0.02	199 (11.4)	<0.01
	Not married	1213 (41.0)	94 (7.8)		205 (16.9)	
Poor self-rated health	No	2559 (86.4)	146 (5.7)	<0.01	NA	
	Yes	404 (13.6)	45 (11.1)		NA	

*Abbreviation: NA* Not applicable.

^a^Calculated by chi-squared tests.

Mean, median, range and correlation among area-based indicators used in this study are shown in Table 2. The mean of ALPBD was 6.9 with a range from 0.0 to 50.0%. ALPBD had moderate correlation with area-level unemployment by census (ALUEC) (0.55) and ALDI (0.63). Two combinations of ALUEC and ALDI, and area-level not-home-owner by aggregated responses (ALNHA) and area-level not-home-owner by census (ALNHC) had an especially high correlation of 0.90 and 0.80, respectively. The distribution of ALPBD was consistent with our a priori knowledge of PBD in areas such as *Buraku* or *Nishinari* except for the low ALPBD areas where only one or two subjects answered “yes” to PBD (data not shown) [16].

**Table 2:** Mean (SD), median, range and correlation matrix of the area-level Indicators among 100 tracts, before making quartiles

**Area-level indicators**		**Mean (SD)**	**Median**	**Min**	**Max**	**Pearson correlation coefficients**					
						***i***	***ii***	***iii***	***iv***	***v***	***vi***
*i)*	Area-level place-based discrimination, aggregated^a^ (ALPBD), %	6.9 (9.4)	3.5	0.0	50.0	1	0.17	0.55	0.23	0.21	0.63
*ii)*	Area-level unemployment, aggregated^a^ (ALUEA), %	5.3 (4.6)	4.4	0.0	18.5		1	0.34	−0.03	−0.02	0.30
*iii)*	Area-level unemployment, census^b^ (ALUEC), %	11.4 (4.0)	11.1	3.0	28.6			1	0.27	0.36	0.90
*iv)*	Area-level not-home-owner, aggregated^a^ (ALNHA), %	44.1 (20.6)	40.0	8.6	100.0				1	0.80	0.43
*v)*	Area-level not-home-owner, census^b^ (ALNHC), %	57.2 (18.4)	55.6	18.5	97.0					1	0.51
*vi)*	Area-level deprivation index, census^b^ (ALDI), score	34.9 (18.7)	31.9	0.0	100.0						1

*Abbreviations: SD* standard deviation, *Min* minimum, *Max* maximum.

^a^The term “aggregated” means area-level aggregates (%) of survey positive responses for individual-level place-based discrimination, unemployment or not-home-owner within each tract.

^b^The term “census” means that area-level indicators were created from the information from Japanese census 2005.

Table 3 shows the proportions of perceived PBD and poor SRH according to the quartiles of area-level indicators with their ranges. Participants who lived in disadvantaged places (higher quartiles) were more likely to answer “yes” to experiencing PBD. The proportion of poor SRH significantly differed by all area-level indicators except for ALNHC.

**Table 3:** Perceived place-based discrimination and poor self-rated health according to the quartile of the area-level indicators

		**Subjects**	**Individual-level place-based discrimination, yes**		**Poor self-rated health**	
**Area-level indicators**	**Quartile (range)**	**N (%)**	**N (%)**	***P *** **for difference**^**a**^	**N (%)**	***P *** **for difference**^**a**^
Area-level place-based discrimination, aggregated^b^	Lowest (0.0-0.0)	869 (29.3)	0 (0.0)	<0.01	91 (10.5)	<0.01
(ALPBD)	2nd (2.7-3.3)	632 (21.3)	19 (3.0)		82 (13.0)	
	3rd (3.4-7.4)	694 (23.4)	34 (4.9)		98 (14.1)	
	Highest (7.7-50.0)	768 (25.9)	138 (18.0)		133 (17.3)	
Area-level unemployed, aggregated^b^	Lowest (0.0-2.3)	728 (24.6)	49 (6.7)	0.01	111 (15.3)	0.01
(ALUEA)	2nd (2.6-3.8)	744 (25.1)	30 (4.0)		97 (13.0)	
	3rd (4.0-8.0)	716 (24.2)	49 (6.8)		75 (10.5)	
	Highest (8.1-18.5)	775 (26.2)	63 (8.1)		121 (15.6)	
Area-level unemployed, census^c^	Lowest (3.0-8.4)	729 (24.6)	23 (3.2)	<0.01	96 (13.2)	<0.01
(ALUEC)	2nd (8.6-10.3)	765 (25.8)	30 (3.9)		88 (11.5)	
	3rd (10.8-13.1)	723 (24.4)	50 (6.9)		91 (12.6)	
	Highest (13.1-28.6)	746 (25.2)	88 (11.8)		129 (17.3)	
Area-level not-home-owner, aggregated^b^	Lowest (8.6-26.9)	737 (24.9)	32 (4.3)	<0.01	76 (10.3)	<0.01
(ALNHA)	2nd (27.3-37.5)	707 (23.9)	46 (6.5)		92 (13.0)	
	3rd (39.3-57.1)	774 (26.1)	48 (6.2)		118 (15.3)	
	Highest (57.9-100.0)	745 (25.1)	65 (8.7)		118 (15.8)	
Area-level not-home-owner, census^c^	Lowest (18.5-43.2)	738 (24.9)	30 (4.1)	<0.01	88 (11.9)	0.17
(ALNHC)	2nd (43.6-54.4)	734 (24.8)	43 (5.9)		92 (12.5)	
	3rd (54.4-68.5)	755 (25.5)	61 (8.1)		113 (15.0)	
	Highest (68.9-97.0)	736 (24.8)	57 (7.7)		111 (15.1)	
Area-level deprivation index, census^c^	Lowest (0.0-19.9)	751 (25.4)	25 (3.3)	<0.01	84 (11.2)	<0.01
(ALDI)	2nd (20.5-31.0)	736 (24.8)	18 (2.5)		98 (13.3)	
	3rd (31.0-46.0)	731 (24.7)	43 (5.9)		81 (11.1)	
	Highest (46.7-100.0)	745 (25.1)	105 (14.1)		141 (18.9)	

Note: Higher quartiles indicate more disadvantaged area.

^a^Calculated by chi-squared tests.

^b^The term “aggregated” means area-level aggregates (%) of survey positive responses for individual-level place-based discrimination, unemployment or not-home-owner within each tract.

^c^The term “census” means that area-level indicators were created from the information from Japanese census 2005.

Tables 4 and 5 show the results of the multilevel logistic analyses. All analyzed individual-level factors showed significant associations with poor SRH in the age, sex, PBD and SES-adjusted model (model 4). For example, ORs (95%CI) were 1.89 (1.33, 2.81), 2.28 (1.50, 3.42) and 1.83 (1.45, 2.30) for perceived PBD, unemployment and not-home-owner, respectively (Table 4). Regarding associations between area-level indicators and poor SRH (Table 5), only area-level indicators were included in model 1. The ORs for the third and highest quartiles of ALPBD, ALNHA and ALNHC, third quartile of area-level unemployment by aggregated responses (ALUEA), and highest quartile of ALDI were statistically significant compared with the lowest quartile: for example, ORs for the highest quartiles of ALPBD, ALNHA, ALNHC and ALDI were 1.84 (1.35, 2.52), 1.57 (1.14, 2.20), 1.65 (1.18, 2.33) and 1.78 (1.30, 2.44), respectively. Results from an age and sex-adjusted model (model 2) did not vary compared with those from model 1. After adjustment for age, sex and a corresponding individual-level factor (model 3), the ORs for highest quartile of ALPBD, ALNHC and ALDI were attenuated, but remained statistically significant: i.e., 1.57 (1.13, 2.18), 1.53 (1.11, 2.12) and 1.66 (1.20, 2.28), respectively. In the age, sex, individual-level PBD and SES-adjusted model (model 4), the ORs for the third and highest quartiles of ALPBD were marginally significant: 1.31 (95%CI: 0.93, 1.84) and 1.32 (0.95, 1.86), respectively, while those of the highest quartiles of ALNHC and ALDI were attenuated to 1.26 and 1.21, respectively, and not significant. In the further social relationship-adjusted model (model 5), the ORs for the highest quartile of ALPBD, ALNHA, ALNHC and ALDI were attenuated to 1.28, 1.10, 1.25 and 1.13, respectively, and all were non-significant, although the ORs for the third quartile of ALUEC were 0.74 and marginally significant. ORs for the third quartile of ALUEA retained significance throughout all models: i.e., 0.65 (0.46, 0.93) for model 1, 0.64 (0.45, 0.90) for model 2, 0.60 (0.42, 0.85) for model 3, 0.65 (0.46, 0.92) for model 4 and 0.67 (0.47, 0.95) for model 5.

**Table 4:** Multilevel odds ratios (95%CI) for poor self-rated health, area-level PBD model

		**Model 1**		**Model 2**		**Model 3**		**Model 4**	
		**Unadjusted model**		**Age- and sex-adjusted model**		**Age, sex and corresponding individual-level factor-adjusted model**^**a**^		**Age, sex, PBD and SES-adjusted model**^**b**^	
		**ORs**	**95%CI**	**ORs**	**95%CI**	**ORs**	**95%CI**	**ORs**	**95%CI**
*Fixed effects*									
Area-level PBD, aggregated^c^	Lowest (reference)	1.00		1.00		1.00		1.00	
(ALPBD)	2nd	1.26	0.89, 1.77	1.23	0.85, 1.74	1.21	0.85, 1.72	1.18	0.83, 1.68
	3rd	1.43	1.03, 2.00	1.42	1.01, 1.99	1.38^d^	0.99, 1.92	1.31^d^	0.93, 1.84
	Highest	1.84	1.35, 2.52	1.76	1.29, 2.43	1.57	1.13, 2.18	1.32^d^	0.95, 1.86
Age group	25-34 (reference)			1.00		1.00		1.00	
	35-44			1.35^d^	0.96, 1.92	1.35^d^	0.96, 1.92	1.36^d^	0.96, 1.95
	45-54			1.86	1.31, 2.65	1.83	1.29, 2.61	2.06	1.44, 2.95
	55-65			2.20	1.59, 3.08	2.22	1.61, 3.11	1.86	1.32, 2.66
Sex	Male (reference)			1.00		1.00		1.00	
	Female			0.74	0.59, 0.91	0.72	0.58, 0.89	0.61	0.48, 0.76
Individual-level perceived PBD	No (reference)					1.00		1.00	
	Yes					1.79	1.21, 2.62	1.89	1.33, 2.81
Working status	Working (reference)							1.00	
	Not working							2.74	2.08, 3.59
	Unemployed							2.28	1.50, 3.42
Housing tenure	Home owner (reference)							1.00	
	Not home owner							1.83	1.45, 2.30
Education attainment	College or more (reference)							1.00	
	High school or less							1.44	1.14, 1.81
*Random effects*									
Area-level variance^e^		0.047		0.044		0.040		0.036	
Deviance information criterion^f^		2301.4		2273.0		2266.7		2170.8	

*Abbreviations: CI* credible interval, *ORs* Odds ratios, *PBD* place-based discrimination, *SES* socioeconomic status.

^a^Individual-level perceived PBD was adjusted as a corresponding individual-level factor in this case in addition to age and sex.

^b^Age, sex, individual-level perceived PBD, working status, housing tenure and education attainment were adjusted.

^c^The term “aggregated” means area-level aggregates (%) of survey positive responses for individual-level place-based discrimination within each tract.

^d^Statistical significance of *P* < 0.1 (marginal significance).

^e^Area-level variance means the extent of variability between areas after fixed effects adjustments.

^f^Deviance information criterion was used to compare the goodness-of-fit of each model. Generally, lower estimates means good fit.

**Table 5:** Associations between area-level indicators and poor SRH determined by multilevel logistic regression

		**Model 1**		**Model 2**		**Model 3**		**Model 4**	
		**Unadjusted model**		**Age- and sex-adjusted model**		**Age, sex and corresponding individual-level factor-adjusted model**^**a**^		**Age, sex, PBD and SES-adjusted model**^**b**^	
***Area-level indicators***		**ORs**	**95%CI**	**ORs**	**95%CI**	**ORs**	**95%CI**	**ORs**	**95%CI**
Area-level PBD, aggregated^c^	Lowest (reference)	1.00		1.00		1.00		1.00	
(ALPBD)^e^	2nd	1.26	0.89, 1.77	1.23	0.85, 1.74	1.21	0.85, 1.72	1.18	0.83, 1.68
	3rd	1.43	1.03, 2.00	1.42	1.01, 1.99	1.38^f^	0.99, 1.92	1.31^f^	0.93, 1.84
	Highest	1.84	1.35, 2.52	1.76	1.29, 2.43	1.57	1.13, 2.18	1.32^f^	0.95, 1.86
Area-level unemployed, aggregated^c^	Lowest (reference)	1.00		1.00		1.00		1.00	
(ALUEA)	2nd	0.83	0.60, 1.16	0.80	0.57, 1.11	0.80	0.56, 1.11	0.84	0.61, 1.16
	3rd	0.65	0.46, 0.93	0.64	0.45, 0.90	0.60	0.42, 0.85	0.65	0.46, 0.92
	Highest	1.06	0.78, 1.46	1.02	0.75, 1.40	0.92	0.66, 1.27	0.90	0.67, 1.23
Area-level unemployed, census^d^	Lowest (reference)	1.00		1.00		1.00		1.00	
(ALUEC)	2nd	0.86	0.62, 1.22	0.85	0.61, 1.20	0.83	0.58, 1.17	0.82	0.59, 1.16
	3rd	0.94	0.67, 1.32	0.92	0.65, 1.29	0.87	0.61, 1.25	0.77	0.55, 1.08
	Highest	1.36	0.99, 1.89	1.32	0.96, 1.81	1.23	0.88, 1.71	0.99	0.72, 1.38
Area-level not-home-owner, aggregated^c^	Lowest (reference)	1.00		1.00		1.00		1.00	
(ALNHA)	2nd	1.28	0.91, 1.82	1.28	0.91, 1.81	1.17	0.82, 1.65	1.16	0.81, 1.65
	3rd	1.53	1.10, 2.13	1.59	1.14, 2.22	1.33	0.94, 1.86	1.34	0.94, 1.89
	Highest	1.57	1.14, 2.20	1.63	1.17, 2.28	1.16	0.82, 1.66	1.13	0.79, 1.63
Area-level not-home-owner, census^d^	Lowest (reference)	1.00		1.00		1.00		1.00	
(ALNHC)	2nd	1.24	0.87, 1.76	1.17	0.82, 1.68	1.18	0.84, 1.65	1.05	0.74, 1.48
	3rd	1.48	1.05, 2.11	1.43	1.02, 2.05	1.50	1.07, 2.08	1.22	0.87, 1.73
	Highest	1.65	1.18, 2.33	1.61	1.14, 2.30	1.53	1.11, 2.12	1.26	0.91, 1.76
Area-level deprivation index, census^d^	Lowest (reference)	1.00		1.00		1.00		1.00	
(ALDI)	2nd	1.16	0.83, 1.61	1.19	0.86, 1.66	1.17	0.83, 1.66	1.09	0.76, 1.53
	3rd	0.96	0.68, 1.35	0.98	0.69, 1.38	0.90	0.62, 1.27	0.82	0.57, 1.15
	Highest	1.78	1.30, 2.44	1.76	1.30, 2.40	1.66	1.20, 2.28	1.21	0.86, 1.68

^a^Working status was adjusted for the model for area-level deprivation index.

^b^Age, sex, individual-level perceived PBD, working status, housing tenure and education attainment were adjusted.

^c^The term “aggregated” means area-level aggregates (%) of survey positive responses for individual-level place-based discrimination, unemployment or not-home-owner within each tract.

^d^The term “census” means that area-level indicators were created from the information from Japanese census 2005.

^e^Transferred from Table 4.

^f^Statistical significance of *P* < 0.1 (marginal significance).

## Discussion

Our results showed that not only individualized PBD but also institutionalized PBD might be social determinants of health after individual-level factors such as SES had been taken into account, although the health impact of institutionalized PBD was not fully statistically significant and was lower than that of the individualized PBD. In our previous study, the association between individual-level PBD and poor mental health was observed after adjustment for SES such as employment, housing tenure and education, and stronger among the highly educated than among the less educated [16], although low education levels were positively associated with poor health in general [28]. In a study in New Zealand, health differences between ethnic minorities and majorities were considerably attenuated after adjustment for perceived discrimination in addition to SES [31]. These two results indicated that individual-level discrimination may affect health independently of SES, consistent with the results in the current study.

In the individual-level PBD and SES-adjusted model (model 4), the ORs of ALPBD for poor SRH were marginally significant, those of other area-level SES indicators were attenuated (i.e., ALNHC and ALDI) and did not show consistent tendency of quartile order (i.e., ALUEA, ALUEC and ALNHA). The lack of hierarchical association between area-level unemployment and poor health was unexpected, because unemployment is a key social factor in major deprivation indices used worldwide [5,29,32]. Because ALUEA was derived from the proportion of an average of 1.6 unemployed persons, ranging between zero and five, in tracts which consist of an average of 29.6 persons, this might be unstable and biased (Additional file 1: Table S1), although ALUEA and ALUEC indicated similar results in model 4 (key model). Because the proportion of unemployed persons within the area is generally low, area-level unemployment may not have wide range of distribution or strong relationship with residents’ health. Thus, the magnitude of area-level indicators should be compared carefully as the ranges of these measurements should be different. As this is conducted in Japan where severe poverty and areal segregation based on living standards are not highly likely, the range of areal deprivation may be narrower than those in other places with wider social disparities such as the USA. The patterning of gradients in health detected by the area-level indicators may reflect both the different meanings of the areas investigated and the different pathways by which diverse aspects of SES (PBD) influence health [5,33].

There is no consensus on an optimal scale of perceived discrimination [34], and various patterns of questions have been used to assess self-reported racial/ethnic discrimination. A single item scale for self-reported discrimination has often been used, but may be unreliable and may understate the extent of discrimination [35], although these problems tend to bias associations between self-reported discrimination and health toward the null (i.e., type II error) [19]. In our results, the distribution of ALPBD was consistent with our a priori knowledge of PBD in areas such as *Buraku* or *Nishinari*. Although our single question about perceived discrimination has not been validated, the fact that our results matched with a priori knowledge may imply an acceptable validity of the scale used for perceived PBD in the study.

### Mechanism between PBD and health

In a previous study, Pearce has suggested that, in terms of the mechanism between PBD and health, individualized and institutionalized five pathways are mutually non-exclusive [20]. First, deteriorated identity or social relations deriving from internalization of PBD have etiological links, particularly with health related behaviors and mental health. Second, a number of life chances, such as education, training opportunities, employment prospects and developing interpersonal relationships, are harmed by the baggage of “moral inferiority” that is associated with residents of highly stigmatized neighborhoods. Thus, consequential relative material deprivation can harm health due to the psychosocial harm of individuals. Third, highly stigmatized areas suffer from disinvestment in environmental goods such as housing, local infrastructure and services, which destabilizes efforts to sustain the social determinants of health; i.e., environmental injustice affects health [36]. Fourth, not only individual social networks but also neighborhood social bonds and collective efficacy are undermined in highly marginalized hidden neighborhoods in response to perceived threats related to PBD. Last, stigmatized areas have been made historically and economically static, because of migration of the poverty and reproduction of the discrimination, and continued deterioration of residents’ health. Although little is known about mechanism between PBD and health, the above mechanism is almost identical to the pathways which have been suggested in the setting of general discrimination, such as racial/ethnic discrimination [18].

### Institutionalized discrimination

According to the above five mechanisms, as the direct impact of interpersonal discrimination is likely to only partially account for the association between PBD and health [20], PBD must be understood and assessed within what Williams et al. describe as “the larger context of institutionalized discrimination [racism] which has created differential exposure to a broad range of stressors” [34]. Institutionalized discrimination due to living in a stigmatized place may be understood as invisible and unconscious exposure such as lack of local environmental investment, suppression of job opportunities and denial of friendships, which can be associated with deterioration of housing conditions, unemployment and poor social relationships, respectively [18,37]. Thus, key social determinants of health such as unemployment and low educational level might not be confounding factors, but mediated factors, between PBD and health. When only a small number of individual social factors are adjusted, neighborhood indicators may be more likely to act as proxies for unmeasured individual-level information. In general, studies adjusting for more individual level SES factors found the extent of the association between area-level SES and health was smaller [38]. On the other hand, controlling for individual SES may remove part of the contextual impact [38]. Therefore, with respect to our findings, the impact of institutionalized PBD on health should be evaluated within the range of models 3 and 5. The non-significant result of ALPBD for poor SRH in model 5 (after adjustment for mediators) might be an underestimation, whereas the statistical significance of ALPBD in model 3 might be an overestimation.

ALPBD may include the traditional spatial pattern in Osaka city [39], corresponding to the fifth mechanism above [20]. It has long been recognized in sociology, geography and economics that the geographic distribution of households is not random but arises from political, economic, historical, and social processes. Our findings accord with the suggestion put forward by Messer, that the neighborhood-forming process cause differential locations of populations and these populations will share the same influences on their health which, in turn, will lead to geographical clusters [40].

### Consideration for not only the bottom layer of society but also the entire population

By using measures of clustering of individual health status within neighborhoods, we were able to evaluate the importance of the neighborhood-level approach [41]. These considerations are important when attempting to determine an approach based on places rather than on individuals. Focusing on a small group in a disadvantaged area may not be an efficient approach as only a small variation in health will be seen and many at-risk people residing outside the disadvantaged area will be ignored [41]. In this study, we focused on the whole range of the population in Osaka city and found the association between poor SRH not only in a priori-considered stigmatized areas (the bottom quartile) but also subsequently stigmatized areas (third quartiles of ALPBD). This suggests that not only is a high-risk population approach necessary for the bottom quartile but that an entire population-based strategy would improve population health [42].

### Limitations

There are several limitations to this study. First, as it is cross-sectional, causal interpretations of the results cannot be established. Whereas the disadvantaged place might damage an individual’s health, those who reported poor SRH might be more likely to move into the disadvantaged area. Furthermore, there may be a common tendency when respondents rated their experience of discrimination and subjective health, which may lead to overestimation of the association [43]. On the other hand, peer comparison, in which lower SES respondents may compare themselves to their relatively unhealthy peers, may lead to relative health “optimism” among lower SES individuals and cause an underestimation of the association [43]. Second, regarding area-level indicators, there are issues of same-source bias and subjective bias [40]. Averaging subjective responses (into quartiles) may be likely to reduce the magnitude of subjective bias in the data. Using census data, rather than an aggregate of study participants, may circumvent the problem of same-source bias. Furthermore, because two area-level indicators of place-based discrimination (key factor in the study) and deprivation were derived from different data sources of the survey and census 2005, respectively, we were afraid that there was merely a difference of data source in these results. We compared the results from the two data sources for area-level unemployment and not-home-owner, because these measures were available in both the survey and census 2005. As the results of multilevel logistic regression (Table 5), discrepancies in results between survey aggregates and census-based indicators were small for area-level not-home-owner, and were relatively small in adjusted model 4 (key model) for area-level unemployment. Therefore, we considered that there might be possible comparability between six area-level indicators derived from the survey aggregates or census 2005. Third, neighborhood characteristics drawn from other aspects (e.g., income inequality, social capital and so on) may be more associated with health than ALPBD, but these relationships will be examined in future research.

## Conclusion

Institutionalized PBD may represent an aspect of place which has a greater impact on health than other area-level SES indicators such as ALDI, after adjustment for individual-level factors, in Osaka, Japan. The role of ALPBD in explaining spatial gradients in health may be smaller than that of individualized PBD. However, even if all interpersonal discrimination is eliminated, the institutionalized structures that shape people’s opportunities in life from early childhood may be so powerful that we should continue to investigate the association between ALPBD and health.

## Competing interests

The authors declare that they have no competing interests.

## Authors’ contributions

TT, WF, IM, EK and HF conceived the study. TT was responsible for data management and statistical analyses. All authors were responsible for the interpretation of the data. TT wrote the report and all authors read and approved the final manuscript.

## Pre-publication history

The pre-publication history for this paper can be accessed here:

http://www.biomedcentral.com/1471-2458/14/449/prepub

## Supplementary Material

Additional file 1 **Deprivation index. Table S1.** Area-level indicators in selected 100 census tracts. **Table S2.** Univariate-adjusted multilevel logistic regression according to basic characteristics. **Tables S3-S7.** Multilevel logistic regression using one of the area-level indicators unemployment (aggregated and census-based), not-home-owner (aggregated and census-based) and deprivation index (census-based). **Table S8.** multilevel logistic regression including further social relationship-adjusted model (model 5). Click here for file
