# Supplementary material for: Individualized and institutionalized residential place-based discrimination and self-rated health: a cross-sectional study of the working-age general population in Osaka city, Japan
Source: BMC Public Health. 2014 May 13;14:449. doi: 10.1186/1471-2458-14-449 (PMC4046154; doi:10.1186/1471-2458-14-449)
Supplement: Additional file 1 — Deprivation index. Table S1. Area-level indicators in selected 100 census tracts. Table S2. Univariate-adjusted multilevel logistic regression according to basic characteristics. Tables S3-S7. Multilevel logistic regression using one of the area-level indicators unemployment (aggregated and census-based), not-home-owner (aggregated and census-based) and deprivation index (census-based). Table S8. multilevel logistic regression including further social relationship-adjusted model (model 5). [file 1471-2458-14-449-S1.docx]

**Additional file 1 (Online Only)**

**Title**

Individualized and institutionalized residential place-based discrimination and self-rated health: a cross-sectional study of the working-age general population in Osaka city, Japan

**Supplementary methods**

***Deprivation index***

The deprivation index of small areas [1] was used as a composite indicator of census variables in order to capture the geographical accumulation of the deprived population living in an area. The index was based on Gordon’s method [2] which was originally designed for directly estimating the population of poverty in a ward in the UK and subsequently used to provide a finer geographical scale and assessing historical changes in poverty concentrations [3, 4]. The method firstly uses a micro dataset with samples of a nationally representative population to predict ‘poverty’ households in logistic regression analysis. It uses deprivation related common variables as covariates that are available both in the micro data and the census tables. Then Gordon’s index of area deprivation takes the form of weighted sum of census-based variables where the weights are given by the estimated odds ratios in the logistic regression analysis using the micro data. The core ‘poverty’ household used for constructing the Japanese deprivation index is operationalized as households satisfying both objective and subjective measures of low socioeconomic position: the equivalized annual household income is below half of the national median; and the surveyed person in the household has low social class identification (bottom two of top-bottom self-placement on a 5-point scale). These two individual socioeconomic status measures are sensitive to self-rate health of the micro data [5]. The derived area-level deprivation index, ALDI, is calculated as:

ALDI = *k* (2.99 * proportion of old couple households + 7.57 * proportion of old single households + 17.4 * proportion of lone mother households + 2.22 * proportion of rent houses + 4.03 * proportion of sales and service workers + 6.05 * proportion of agricultural workers + 5.38 * proportion of blue-collar workers + 18.3 * unemployment rate),

where *k* is a balancing factor which should be a positive constant. Gordon assumed that the weighted sum of census variables is proportional to the rate of ‘poverty’ household in an area and proposed computing *k* to satisfy the condition that the national poverty rate as the average of ALDI weighted by household numbers of areas is equal to the estimated number of poverty rate based on the nationally representative micro dataset. Since only the relative position of small areas within the Osaka city is needed for this study, we simply standardized the ALDI score ranging from 0 to 100. The index shows consistent positive associations with all-cause and various cancer mortalities at the municipality level in Japan. The weights used here are slightly different from those used in the original paper [1] due to some minor modification of occupational categories and missing cases in the micro data but there is no essential difference in scores.

**Supplementary results**

The distribution of selected area-level indicators used in the study is shown in Table S1 according to selected 100 census tracts.

Results of the univariate-adjusted multilevel logistic regression for individual-level place-based discrimination (PBD) and poor self-rated health according to basic characteristics are shown in Table S2. The results of the multilevel logistic regression using one of the area-level indicators unemployment (aggregated and census-based), not-home-owner (aggregated and census-based) and deprivation index (census-based) are shown in Table S3-S7, respectively. Summing these results for area-level indicators and poor SRH, the results of the multilevel logistic regression including further social relationship-adjusted model (model 5) are shown in Table S8 (Table 5 plus model 5).

**Supplementary references**

1. Nakaya T: **Evaluating Socio-economic Inequalities in Cancer Mortality by Using Areal Statistics in Japan: A Note on the Relation between Municipal Cancer Mortality and Areal Deprivation Index**. *Proceedings of the Institute of Statistical Mathematics* 2011, **59**(2):239-265. (in Japanese)

2. Gordon D: **Census based deprivation indices: their weighting and validation**. *J Epidemiol Community Health* 1995, **49 Suppl 2**:S39-44.

3. Fahmy E, Gordon D, Dorling D, Rigby J, Wheeler B: **Poverty and place in Britain, 1968-99**. *Environment and Planning A* 2011, **43**(3):594-617.

4. Saunders J: **Weighted Census-based deprivation indices: their use in small areas**. *J Public Health Med* 1998, **20**(3):253-260.

5. Hanibuchi T, Nakaya T, Murata C: **Socio-economic status and self-rated health in East Asia: a comparison of China, Japan, South Korea and Taiwan**. *European journal of public health* 2012, **22**(1):47-52.

| Table S2. Results of Univariate-adjusted Multilevel Logistic Regression for Individual-level Place-based Discrimination and Poor Self-rated Health According to Basic Characteristics | | | | |
| --- | --- | --- | --- | --- |
|  |  | For Individual-level  Place-based Discrimination |  | For Poor Self-rated Health |
| Characteristics |  | Odds Ratios (95% CI) |  | Odds Ratios (95% CI) |
| Sex | Male | 1.00 |  | 1.00 |
|  | Female | 2.05 (1.48, 2.89) |  | 0.73 (0.61, 0.90) |
| Age group | 25-34 years | 1.00 |  | 1.00 |
|  | 35-44 years | 1.13 (0.70, 1.82) |  | 1.39 (0.99, 2.00) |
|  | 45-54 years | 1.64 (1.04, 2.65) |  | 1.99 (1.41, 2.86) |
|  | 55-65 years | 0.93 (0.57, 1.50) |  | 2.40 (1.72, 3.41) |
| Perceived place-based discrimination | No | NA |  | 1.00 |
|  | Yes | NA |  | 2.01 (1.39, 2.87) |
| Working status | Working | 1.00 |  | 1.00 |
|  | Not working | 0.99 (0.64, 1.49) |  | 2.50 (1.95, 3.20) |
|  | Unemployed | 1.19 (0.61, 2.23) |  | 2.59 (1.73, 3.83) |
| Housing tenure | Home owner | 1.00 |  | 1.00 |
|  | Not home owner | 0.80 (0.57, 1.13) |  | 1.77 (1.43, 2.19) |
| Education attainment | College or more | 1.00 |  | 1.00 |
|  | High school or less | 1.14 (0.83, 1.58) |  | 1.85 (1.49, 2.30) |
| Number of friends | 0 | 1.00 |  | 1.00 |
|  | 1-4 | 2.76 (1.29, 6.48) |  | 0.52 (0.38, 0.71) |
|  | 5 or more | 3.71 (1.74, 8.63) |  | 0.34 (0.25, 0.46) |
| Marital status | Married | 1.00 |  | 1.00 |
|  | Not married | 1.18 (0.85, 1.63) |  | 1.57 (1.27, 1.94) |
| Poor self-rated health | No | 1.00 |  | NA |
|  | Yes | 1.79 (1.20, 2.62) |  | NA |
| Abbreviation: NA; Not applicable |  |  |  |  |

| Table S8. Associations Between Area-level Indicators and Poor SRH Determined by Multilevel Logistic Regression | | | | | | | | | | | | | | | | |
| --- | --- | --- | --- | --- | --- | --- | --- | --- | --- | --- | --- | --- | --- | --- | --- | --- |
|  |  | Model 1 | |  | Model 2 | |  | Model 3 | |  | Model 4 | |  | Model 5 | | |
|  |  | Unadjusted Model | |  | Age- and Sex-adjusted Model | |  | Age, Sex and Corresponding Individual-level Factor-adjusted Model^a^ | |  | Age, Sex, PBD and SES-adjusted Model | |  | Model 4 + Social Relationship-adjusted Model | | |
| *Area-level Indicators* |  | ORs | 95%CI |  | ORs | 95%CI |  | ORs | 95%CI |  | ORs | 95%CI |  | ORs | 95%CI | |
| Area-level PBD, aggregated^b^ | Lowest (reference) | 1.00 |  |  | 1.00 |  |  | 1.00 |  |  | 1.00 |  |  | 1.00 |  | |
| (ALPBD) | 2nd | 1.26 | 0.89, 1.77 |  | 1.23 | 0.85, 1.74 |  | 1.21 | 0.85, 1.72 |  | 1.18 | 0.83, 1.68 |  | 1.23 | 0.86, 1.75 | |
|  | 3rd | 1.43 | 1.03, 2.00 |  | 1.42 | 1.01, 1.99 |  | 1.38 ^d^ | 0.99, 1.92 |  | 1.31 ^d^ | 0.93, 1.84 |  | 1.29 | 0.92, 1.81 | |
|  | Highest | 1.84 | 1.35, 2.52 |  | 1.76 | 1.29, 2.43 |  | 1.57 | 1.13, 2.18 |  | 1.32 ^d^ | 0.95, 1.86 |  | 1.28 | 0.92, 1.79 | |
| Area-level unemployed, aggregated^b^ | Lowest (reference) | 1.00 |  |  | 1.00 |  |  | 1.00 |  |  | 1.00 |  |  | 1.00 |  | |
| (ALUEA) | 2nd | 0.83 | 0.60, 1.16 |  | 0.80 | 0.57, 1.11 |  | 0.80 | 0.56, 1.11 |  | 0.84 | 0.61, 1.16 |  | 0.89 | 0.64, 1.23 | |
|  | 3rd | 0.65 | 0.46, 0.93 |  | 0.64 | 0.45, 0.90 |  | 0.60 | 0.42, 0.85 |  | 0.65 | 0.46, 0.92 |  | 0.67 | 0.47, 0.95 | |
|  | Highest | 1.06 | 0.78, 1.46 |  | 1.02 | 0.75, 1.40 |  | 0.92 | 0.66, 1.27 |  | 0.90 | 0.67, 1.23 |  | 0.91 | 0.66, 1.25 | |
| Area-level unemployed, census^c^ | Lowest (reference) | 1.00 |  |  | 1.00 |  |  | 1.00 |  |  | 1.00 |  |  | 1.00 |  | |
| (ALUEC) | 2nd | 0.86 | 0.62, 1.22 |  | 0.85 | 0.61, 1.20 |  | 0.83 | 0.58, 1.17 |  | 0.82 | 0.59, 1.16 |  | 0.80 | 0.56, 1.12 | |
|  | 3rd | 0.94 | 0.67, 1.32 |  | 0.92 | 0.65, 1.29 |  | 0.87 | 0.61, 1.25 |  | 0.77 | 0.55, 1.08 |  | 0.74 ^d^ | 0.52, 1.03 | |
|  | Highest | 1.36 | 0.99, 1.89 |  | 1.32 | 0.96, 1.81 |  | 1.23 | 0.88, 1.71 |  | 0.99 | 0.72, 1.38 |  | 0.91 | 0.65, 1.27 | |
| Area-level not-home-owner, aggregated^b^ | Lowest (reference) | 1.00 |  |  | 1.00 |  |  | 1.00 |  |  | 1.00 |  |  | 1.00 |  | |
| (ALNHA) | 2nd | 1.28 | 0.91, 1.82 |  | 1.28 | 0.91, 1.81 |  | 1.17 | 0.82, 1.65 |  | 1.16 | 0.81, 1.65 |  | 1.17 | 0.82, 1.66 | |
|  | 3rd | 1.53 | 1.10, 2.13 |  | 1.59 | 1.14, 2.22 |  | 1.33 | 0.94, 1.86 |  | 1.34 | 0.94, 1.89 |  | 1.31 | 0.93, 1.85 | |
|  | Highest | 1.57 | 1.14, 2.20 |  | 1.63 | 1.17, 2.28 |  | 1.16 | 0.82, 1.66 |  | 1.13 | 0.79, 1.63 |  | 1.10 | 0.76, 1.58 | |
| Area-level not-home-owner, census^c^ | Lowest (reference) | 1.00 |  |  | 1.00 |  |  | 1.00 |  |  | 1.00 |  |  | 1.00 |  | |
| (ALNHC) | 2nd | 1.24 | 0.87, 1.76 |  | 1.17 | 0.82, 1.68 |  | 1.18 | 0.84, 1.65 |  | 1.05 | 0.74, 1.48 |  | 1.04 | 0.73, 1.49 | |
|  | 3rd | 1.48 | 1.05, 2.11 |  | 1.43 | 1.02, 2.05 |  | 1.50 | 1.07, 2.08 |  | 1.22 | 0.87, 1.73 |  | 1.23 | 0.87, 1.75 | |
|  | Highest | 1.65 | 1.18, 2.33 |  | 1.61 | 1.14, 2.30 |  | 1.53 | 1.11, 2.12 |  | 1.26 | 0.91, 1.76 |  | 1.25 | 0.88, 1.79 | |
| Area-level deprivation index, census^c^ | Lowest (reference) | 1.00 |  |  | 1.00 |  |  | 1.00 |  |  | 1.00 |  |  | 1.00 |  | |
| (ALDI) | 2nd | 1.16 | 0.83, 1.61 |  | 1.19 | 0.86, 1.66 |  | 1.17 | 0.83, 1.66 |  | 1.09 | 0.76, 1.53 |  | 1.08 | 0.77, 1.50 | |
|  | 3rd | 0.96 | 0.68, 1.35 |  | 0.98 | 0.69, 1.38 |  | 0.90 | 0.62, 1.27 |  | 0.82 | 0.57, 1.15 |  | 0.80 | 0.56, 1.13 | |
|  | Highest | 1.78 | 1.30, 2.44 |  | 1.76 | 1.30, 2.40 |  | 1.66 | 1.20, 2.28 |  | 1.21 | 0.86, 1.68 |  | 1.13 | 0.81, 1.59 | |
| Abbreviations: CI, credible interval; ORs, Odds ratios; PBD, place-based discrimination; SES, socioeconomic status; SRH, self-rated health. | | | | | | | | | |  |  |  |  |  |  | |
| ^a^Working status was adjusted for the model for area-level deprivation index. | | | | | | | | | | | |  |  |  |  | |
| ^b^The term "aggregated" means area-level aggregates (%) of survey positive responses for individual-level place-based discrimination, unemployment or not-home-owner within each tract. | | | | | | | | | | | | | | | |  |
| ^c^The term "census" means that area-level indicators were created from the information from Japanese census 2005. | | | | | | | | | | | | | | | |  |

^d^Statistical significance of *P*< 0.1 (marginal significance).
